# Prevalence and risk factors of hemodynamic instability associated with preload-dependence during continuous renal replacement therapy in a prospective observational cohort of critically ill patients

**DOI:** 10.1186/s13613-021-00883-9

**Published:** 2021-06-14

**Authors:** Guillaume Chazot, Laurent Bitker, Mehdi Mezidi, Nader Chebib, Paul Chabert, Louis Chauvelot, Laure Folliet, Guillaume David, Judith Provoost, Hodane Yonis, Jean-Christophe Richard

**Affiliations:** 1 Service de Médecine Intensive - Réanimation, Hôpital De La Croix Rousse, Hospices Civils de Lyon, 103 Grande Rue de la Croix Rousse, 69004 Lyon, France; 2 Université de Lyon, Université LYON I, Lyon, France; 3 CREATIS INSERM 1044 CNRS 5220, Villeurbanne, France

**Keywords:** Renal replacement therapy, Acute circulatory failure, Preload-dependence, Pulse contour, Thermodilution, Hemodynamic instability, Acute kidney injury, Net ultrafiltration rate

## Abstract

**Background:**

Hemodynamic instability is a frequent complication of continuous renal replacement therapy (CRRT). Postural tests (i.e., passive leg raising in the supine position or Trendelenburg maneuver in the prone position) combined with measurement of cardiac output are highly reliable to identify preload-dependence and may provide new insights into the mechanisms involved in hemodynamic instability related to CRRT (HIRRT). We aimed to assess the prevalence and risk factors of HIRRT associated with preload-dependence in ICU patients.

We conducted a single-center prospective observational cohort study in ICU patients with acute kidney injury KDIGO 3, started on CRRT in the last 24 h, and monitored with a PiCCO® device. The primary endpoint was the rate of HIRRT episodes associated with preload-dependence during the first 7 days after inclusion. HIRRT was defined as the occurrence of a mean arterial pressure below 65 mmHg requiring therapeutic intervention. Preload-dependence was assessed by postural tests every 4 h, and during each HIRRT episode. Data are expressed in median [1st quartile–3rd quartile], unless stated otherwise.

**Results:**

42 patients (62% male, age 69 [59–77] year, SAPS-2 65 [49–76]) were included 6 [1–16] h after CRRT initiation and studied continuously for 121 [60–147] h. A median of 5 [3–8] HIRRT episodes occurred per patient, for a pooled total of 243 episodes. 131 episodes (54% [CI_95%_ 48–60%]) were associated with preload-dependence, 108 (44%, [CI_95%_ 38–51%]) without preload-dependence, and 4 were unclassified. Multivariate analysis (using variables collected prior to HIRRT) identified the following variables as risk factors for the occurrence of HIRRT associated with preload-dependence: preload-dependence before HIRRT [odds ratio (OR) = 3.82, *p* < 0.001], delay since last HIRRT episode > 8 h (OR = 0.56, *p* < 0.05), lactate (OR = 1.21 per 1-mmol L^−1^ increase, *p* < 0.05), cardiac index (OR = 0.47 per 1-L min^−1^ m^−2^ increase, *p* < 0.001) and SOFA at ICU admission (OR = 0.91 per 1-point increase, *p* < 0.001). None of the CRRT settings was identified as risk factor for HIRRT.

**Conclusions:**

In this single-center study, HIRRT associated with preload-dependence was slightly more frequent than HIRRT without preload-dependence in ICU patients undergoing CRRT. Testing for preload-dependence could help avoiding unnecessary decrease of fluid removal in preload-independent HIRRT during CRRT.

**Supplementary Information:**

The online version contains supplementary material available at 10.1186/s13613-021-00883-9.

## Background

Acute kidney injury (AKI) is independently associated with morbidity and mortality in critically ill patients [1]. A positive fluid balance during AKI is independently associated with mortality in observational studies [2, 3], suggesting that optimizing net ultrafiltration rate to control fluid balance may improve AKI mortality. However, hemodynamic instability related to renal replacement therapy (HIRRT) may be related to excessive fluid removal and may also impair mortality and renal recovery [4–6]. In hemodynamically unstable patients, continuous renal replacement therapy (CRRT) is the preferred modality since it may be associated with better hemodynamic tolerance [7–9]. In clinical practice, the consequence of HIRRT occurrence is often a reduction or discontinuation of the net ultrafiltration rate, while the underlying mechanism of HIRRT may be unrelated to fluid removal. Indeed, HIRRT may be related to the underlying cause of AKI, to cardiac output decrease of various origins (hypovolemia, hypocalcemia, diastolic dysfunction, etc.) or to alterations of the vasomotor tone related to membrane/circuit bio-incompatibility, ultrafiltrate/dialysate temperature or ionic imbalance, among others [10].

We previously showed that most of HIRRT episodes occurring during intermittent hemodialysis are unrelated to preload-dependence (i.e., cardiac output increase in response to fluid administration), and should not necessarily lead to reduction of fluid removal by hemodialysis [11]. To our knowledge, there is no published study reporting the prevalence of HIRRT associated with preload-dependence under CRRT. Since postural tests [i.e., passive leg raising (PLR) in the supine position or Trendelenburg maneuver in the prone position] combined to continuous measurement of cardiac output are highly reliable to identify preload-dependence [12, 13], we hypothesized that their implementation in CRRT monitoring may provide new insights into the mechanisms involved in HIRRT.

## Methods

### Study aim

The aim of this study was to assess prevalence and risk factors of HIRRT associated with preload-dependence during the first 7 days of CRRT.

### Study design and setting

We conducted a prospective, observational, single-center cohort study between May 9, 2017 and September 1st, 2020 in a 15-bed medical intensive care unit (ICU). The study was approved by an ethics committee (CPP Ile de France IV, ID-RCB 2017-A00483-50) and was registered on *ClinicalTrials.gov* (NCT 03139123) on May 2nd, 2017. Informed consent for study inclusion was obtained from all individual participants and/or their closest relatives.

### Patients

To be eligible, the subjects had to fulfill all the following inclusion criteria: aged 18 years or older, with acute kidney injury KDIGO 3 [14], treated with CRRT for less than 24 h and monitored by mean of a PiCCO® device (Pulsion Medical Systems, Feldkirchen, GERMANY) mandated by acute circulatory failure. Exclusion criteria were pregnancy, lower limb amputation, intracranial hypertension, known obstruction of inferior vena cava, ongoing directives to withhold or withdraw life sustaining treatment, lack of consent by patient or next of kin, lack of affiliation to social security, patient under a legal protective measure, inclusion in another research study and previous inclusion in current study.

### Data collection

The following variables were recorded at inclusion: demographic and anthropometric data, time of ICU admission and inclusion, admission category, Sequential Organ Failure Assessment (SOFA) score [15] at ICU admission, and Simplified Acute Physiology Score (SAPS) II at ICU admission [16].

The following variables were recorded at inclusion, every 4 h and at the onset of each HIRRT episode until study completion: heart rate, systolic, diastolic and mean arterial pressures, pulse pressure variation (PPV), central venous pressure, cardiac index assessed by both thermodilution and pulse contour analysis, stroke volume variation (SVV), extravascular lung water index, global end-diastolic volume index, pulmonary vascular permeability index, global ejection fraction, vasopressor administration and dose, inotrope administration, mechanical ventilation use, CRRT settings (blood flow, ultrafiltrate or dialysate rate and temperature, net ultrafiltration rate), and preload-dependency tested as described below.

The following variables were recorded at inclusion and daily until study completion: SOFA score [15], body weight, fluid balance, arterial blood gas, arterial lactate, hemoglobin, fulfillment of sepsis and septic shock criteria [17].

Missing data per variable are reported in Additional file 1.

### Study follow-up

Patients were followed during the first 7 days after inclusion or less in case of occurrence of any of the following events: death, end of life care, CRRT cessation or interruption of PiCCO® monitoring.

### CRRT management

The indication, technique [continuous veno-venous hemofiltration (CVVH) or continuous veno-venous hemodialysis (CVVHD)] and settings of CRRT were under the responsibility of the clinician in charge of the patients, in accordance with current practice guidelines [14]. CRRT was performed with the Multifiltrate® station and the Ultraflux® AV1000S hemofilter (Fresenius Medical Care, Bad Homburg, GERMANY). CRRT settings were adjusted by the attending physician. The ICU policy was to promote hemodynamic monitoring, using the PiCCO® device whenever severe shock was present in patients being treated with CRRT.

### Hemodynamic measurements

HIRRT was defined as mean arterial pressure below 65 mmHg justifying any therapeutic intervention among the following ones: fluid administration, initiation or increase in vasopressor dose, or discontinuation or decrease of net ultrafiltration rate on CRRT. Once hypotension occurred and before any therapeutic intervention, a postural test (PLR in the supine position or Trendelenburg maneuver in the prone position) was performed by trained ICU nurses during 1 min to assess for preload-dependence. PLR was performed from the semi-recumbent position with the trunk at 45° [18] and the Trendelenburg maneuver was performed from a 13° upward bed angulation to a − 13° downward bed angulation in patients in the prone position [13]. Preload-dependence was deemed present if the pulse contour-derived cardiac index increased by at least 10% and 8% during the PLR test and the Trendelenburg maneuver, respectively.

Therapeutic management of HIRRT was at the discretion of the clinician in charge of the patient and was not protocolized. A 1-h period without new hemodynamic assessment was allowed after each HIRRT episode onset to wait for treatment effect.

Hemodynamic measurements including a postural test were systematically performed by trained ICU nurses every 4 h and during each HIRRT episode. Regular training sessions of nurses to hemodynamic measurements were organized to ensure quality of data acquisition. Arterial and central venous blood pressures were continuously monitored, using arterial femoral and jugular vein catheters, respectively, connected to an Intellivue MP40 monitor equipped with the PiCCO® technology module (Philips Healthcare, Andover, MA, USA). Cardiac output was assessed using the PiCCO® device, calibrated with the transpulmonary thermodilution technique at least every 4 h, using a triplicate intravenous infusion of 15 mL cold serum saline. Cardiac output was then continuously monitored using pulse contour analysis with the PiCCO® device. Arterial dynamic elastance was computed as the ratio of PPV over SVV.

### End points

Primary end point was the rate of HIRRT associated with preload-dependence, with reference to the total number of HIRRT episodes occurring during the first 7 days after inclusion. Secondary end point was the identification of risk factors for HIRRT associated with preload-dependence.

### Statistical analysis

Statistical analyses were performed using R software version 4.0.2 [19] and the following packages: lme4 [20], Lmertest [21], pROC [22], PropCIs [23], MultinomialCI [24] and mice [25]. A *p* value below 0.05 was chosen for statistical significance. The statistical unit was the hemodynamic measurement. Power of the study was computed using the normal approximation confidence interval method [26]. Assuming a rate of HIRRT associated with preload-dependence between 0.25 and 0.5, we calculated that with a sample size between 72 and 96 HIRRT episodes, the study would provide at worst a ± 10% precision in the 95% confidence interval of the prevalence of HIRRT associated with preload-dependence. We decided to include conservatively at least 100 HIRRT episodes and at least 50 patients to ensure minimal representativity. Analyses were performed on all included patients, including those prematurely withdrawn. Medians and interquartile ranges were reported for continuous variables and counts in each category with corresponding percentages were reported for categorical variables. Ninety-five percent confidence intervals (CI_95%_) for multinomial proportions were computed using the Sison and Glaz method [27]. To test whether each therapeutic intervention (namely fluid administration, initiation or increase in vasopressor dose, or discontinuation or decrease of net ultrafiltration rate on CRRT) differed between preload-dependent and preload-independent HIRRT episodes, we used 3 logistic regression mixed models with HIRRT type as the dependent variable, each therapeutic intervention as binary independent variable and patient as variable with a random effect, and the Bonferroni correction was used to account for multiple testing. To test which variables could predict occurrence of HIRRT associated with preload-dependence, the whole dataset was restricted to hemodynamic measurements obtained without HIRRT, and a new variable was computed [occurrence of HIRRT associated with preload-dependence in the subsequent measurement (Yes/No)]. Variables were entered into a mixed logistic regression model, using patient as variable with a random effect, and occurrence of HIRRT associated with preload-dependence in the subsequent measurement as the dependent variable. Some continuous variables were entered in the model as dichotomized variables, using ROC curve analysis and computation of optimal cut-off points by maximizing the Youden’s index. Independent variables associated with occurrence of HIRRT with preload-dependence with a p value below 0.2 in univariate analysis were selected for inclusion in a multivariable mixed logistic regression model, using backward stepwise descending selection. Interactions between predictors were assessed on the final model. Missing data in multivariate analyses were handled using multiple imputations and predictive mean matching. Model calibration was assessed by the Hosmer–Lemeshow test and model discrimination by the C-statistic.

## Results

### Screening

**Fig. 1:**
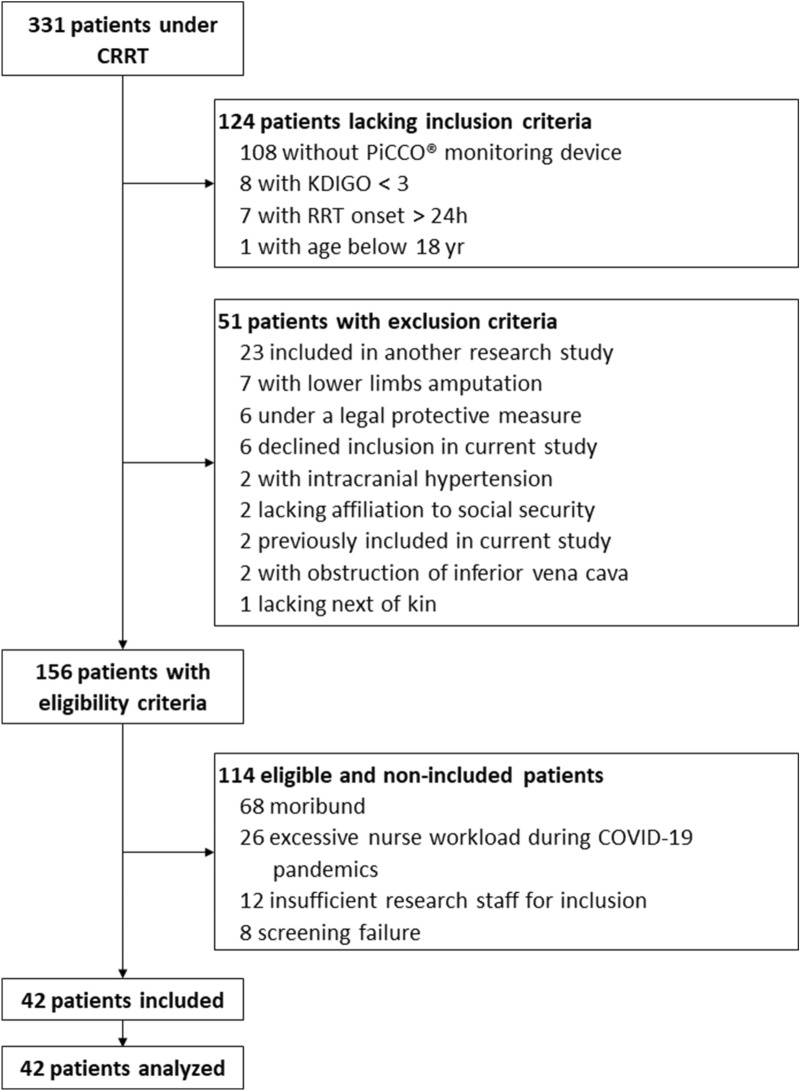
Study flowchart. *CRRT* continuous renal replacement therapy, *RRT* renal replacement therapy

During the study period, 331 patients underwent CRRT and 42 were included. Reasons for non-inclusion are listed in Fig. 1.

### Population characteristics at ICU admission and at inclusion (Table 1)

**Table 1:** Population characteristics at ICU admission and at study inclusion

Variables	Median [IQR] or count (%)
Collected at ICU admission	
Age (year)	69 [59–77]
Male gender	26 (62%)
Admission category	
Medical	41 (98%)
Urgent surgery	1 (2%)
SAPS 2 at ICU admission	65 [49–76]
BMI at ICU admission (kg m^−2^)	26 [22–31]
Comorbidities	
Diabetes	12 (29%)
CRF	4 (10%)
CHF	10 (24%)
Coronary disease	12 (29%)
Cirrhosis	6 (14%)
SOFA score	12 [8–15]
Collected at study inclusion	
Time between ICU admission and CRRT onset	36 [8–73]
Time between CRRT onset and inclusion (h)	6 [1–16]
Sepsis^a^	33 (79%)
Septic shock^a^	24 (57%)
SOFA score	14 [12–16]
Mechanical ventilation	35 (83%)
Vasopressor norepinephrine administration	40 (95%)
Dobutamine administration	6 (14%)
Cumulative fluid balance from ICU admission (kg)	2 [0–6]

*BMI* body mass index, *CHF* chronic heart failure, *CRF* chronic respiratory failure, *CRRT* continuous renal replacement therapy, *ICU* intensive care unit, *IQR* interquartile range, *SAPS* 2 Simplified Acute Physiology Score 2, *SOFA* Sequential Organ Failure Assessment

^a^According to sepsis 3 criteria

Enrollment stopped prematurely after inclusion of 42 patients since nurse staff overwork related to the COVID-19 pandemics hindered inclusions, and since the required number of HIRRT episodes had been substantially exceeded. Forty-two patients (62% male) with median age 69 [59–77] years were included 6 [1–16] h after CRRT onset. At inclusion, 35 patients (83%) underwent mechanical ventilation, 33 (79%) fulfilled sepsis criteria and 24 (57%) fulfilled septic shock criteria [17]. The cumulative fluid balance between admission and inclusion was 2 [0–6] kg.

### Hemodynamic data and CRRT settings at inclusion (Table 2)

**Table 2:** Hemodynamic data and CRRT settings at inclusion

Variables	Median [IQR] or count (%)
Vasopressor norepinephrine dose (µg kg^−1^ min^−1^)	0.54 [0.21–1.41]
Arterial lactate (mmol L^−1^)	2.9 [1.5–5.0]
Heart rate (min^−1^)	96 [74–113]
MAP (mmHg)	70 [62–75]
CVP (mmHg)	8 [6–10]
CI_TD_ (L min^−1^ m^−2^)	2.8 [2.1–3.3]
CI_PC_ (L min^−1^ m^−2^)	2.6 [2.1–3.2]
ISVR (dynes s cm^−5^)	1757 [1277–2224]
EVLWI (mL kg^−1^ PBW)	11.5 [8.9–15.2]
PVPI	2.2 [1.9–3.0]
GEDVI (mL m^−2^)	665 [593–843]
GEF (%)	18 [14–24]
PPV (%)	9 [5–14]
SVV (%)	13 [7–20]
Ea_dyn_	0.8 [0.6–1.0]
Preload-dependence assessed by postural test	22 (52%)
Type of CRRT	
CVVH	39 (93%)
CVVHD	3 (7%)
Ultrafiltration rate (mL kg^−1^ h^−1^)^a^	26 [24–31]
Dialysate rate (mL kg^−1^ h^−1^)^b^	23 [16–27]
CRRT blood flow (mL min^−1^)	250 [200–250]
Net ultrafiltration rate (mL kg^−1^ h^−1^)	0 [0–2.7]
Ultrafiltrate/dialysate temperature (°C)	38 [37–39]
CRRT circuit anticoagulation	
Heparin	39 (93%)
Citrate	3 (7%)

*CRRT* continuous renal replacement therapy, *CI*_*PC*_ cardiac index assessed by pulse contour analysis, *CI*_*TD*_ cardiac index assessed by thermodilution, *CVP* central venous pressure, *CVVH* continuous veno-venous hemofiltration, *CVVHD* continuous veno-venous hemodialysis, *Ea*_*dyn*_ dynamic arterial elastance, *EVLWI* extravascular lung water index, *GEDVI* global end-diastolic volume index, *GEF* global ejection fraction, *IQR* interquartile range, *ISVR* indexed systemic vascular resistance, *MAP* mean arterial pressure, *PBW* predicted body weight, *PPV* pulse pressure variation, *PVPI* pulmonary vascular permeability index, *SVV* stroke volume variation

^a^In patients treated with CVVH

^b^In patients treated with CVVHD

Forty patients (95%) were under norepinephrine with a median dose of 0.54 [0.21–1.41] µg kg^−1^ min^−1^, and preload-dependence was identified in 22 (52%) patients. Thirty-nine patients (93%) underwent CVVH and 3 (7%) were treated by CVVHD. Net ultrafiltration rate amounted to 0 [0–2.7] mL kg^−1^ h^−1^ at inclusion.

### Hemodynamic data and CRRT settings during the study (Table 3)

**Table 3:** Hemodynamic evaluations and CRRT settings during study

Variables	Median [IQR] or count (%)
Number of hemodynamic evaluations	1237
Number of hemodynamic evaluations per patient	33 [19–41]
Study duration (h)	121 [60–147]
Norepinephrine vasopressor dose (µg kg^−1^ min^−1^)	0.29 [0.08–0.81]
Vasopressor norepinephrine administration (%)	1042 (85%)
Dobutamine administration (%)	91 (7%)
Arterial lactate (mmol L^−1^)	1.8 [1.4–2.9]
Heart rate (min^−1^)	94 [78–110]
MAP (mmHg)	73 [64–81]
CVP (mmHg)	7 [4–10]
CI_TD_ (L min^−1^ m^−2^)	3.0 [2.5–3.5]
CI_PC_ (L min^−1^ m^−2^)	2.9 [2.4–3.5]
ISVR (dynes s cm^−5^)	1780 [1507–2095]
EVLWI (mL kg^−1^ PBW)	10.5 [8.7–13.3]
PVPI	2.1 [1.8–2.5]
GEDVI (mL m^−2^)	652 [582–811]
GEF (%)	20 [16–23]
PPV (%)	9 [5–15]
SVV (%)	11 [7–18]
Ea_dyn_	0.8 [0.7–1.0]
Preload-dependence assessed by postural test	490 (41%)
Type of CRRT	
CVVH	1072 (87%)
CVVHD	165 (13%)
Ultrafiltration rate (mL kg^−1^ h^−1^)^a^	27 [24–31]
Dialysate rate (mL kg^−1^ h^−1^)^b^	25 [23–27]
CRRT blood flow (mL min^−1^)	250 [200–250]
Net ultrafiltration rate (mL kg^−1^ h^−1^)	1.4 [0–2.9]
Ultrafiltrate/dialysate temperature (°C)	38 [37–39]
CRRT circuit anticoagulation	
Heparin	946 (77%)
Citrate	165 (13%)
None	126 (10%)

*CI*_*PC*_ continuous cardiac index assessed by pulsed contour analysis, *CI*_*TD*_ cardiac index assessed by thermodilution, *CRRT* continuous renal replacement therapy, *CVP* central venous pressure, *CVVH* continuous veno-venous hemofiltration, *CVVHD* continuous veno-venous hemodialysis, *Ea*_*dyn*_ dynamic arterial elastance, *EVLWI* extravascular lung water index, *GEDVI* global end-diastolic volume index, *GEF* global ejection fraction, *IQR* interquartile range, *MAP* mean arterial pressure, *ISVR* indexed systemic vascular resistance, *PBW* predicted body weight, *PVPI* pulmonary vascular permeability index, *PPV* pulse pressure variation, *PVPI* pulmonary vascular permeability index, *SVV* stroke volume variation

^a^In patients treated with CVVH

^b^In patients treated with CVVHD

Patients were followed during 121 [60–147] h (Additional file 2) with 33 [19–41] hemodynamic evaluations per patient for a total of 1237 hemodynamic evaluations. 28 (67%) patients were prematurely withdrawn from the study (15 due to CRRT cessation, 4 to end of life withdrawal of care, 8 to death before end of study, and 1 to interruption of PiCCO® monitoring).

During the study, norepinephrine was the only vasopressor administered, norepinephrine dose was 0.29 [0.08–0.81] µg kg^−1^ min^−1^, arterial lactate was 1.8 [1.4–2.9] mmol L^−1^. Preload-dependence was present in 41% of the hemodynamic evaluations. CRRT modality was CVVH or CVVHD during 87% and 13% of the hemodynamic evaluations, respectively. Ultrafiltration rate was 27 [24–31] mL kg^−1^ h^−1^ in CVVH-treated patients, dialysate rate was 25 [23–27] mL h^−1^ in CVVHD-treated patients, and net ultrafiltration rate was 1.4 [0–2.9] mL kg^−1^ h^−1^.

### HIRRT episodes

**Fig. 2:**
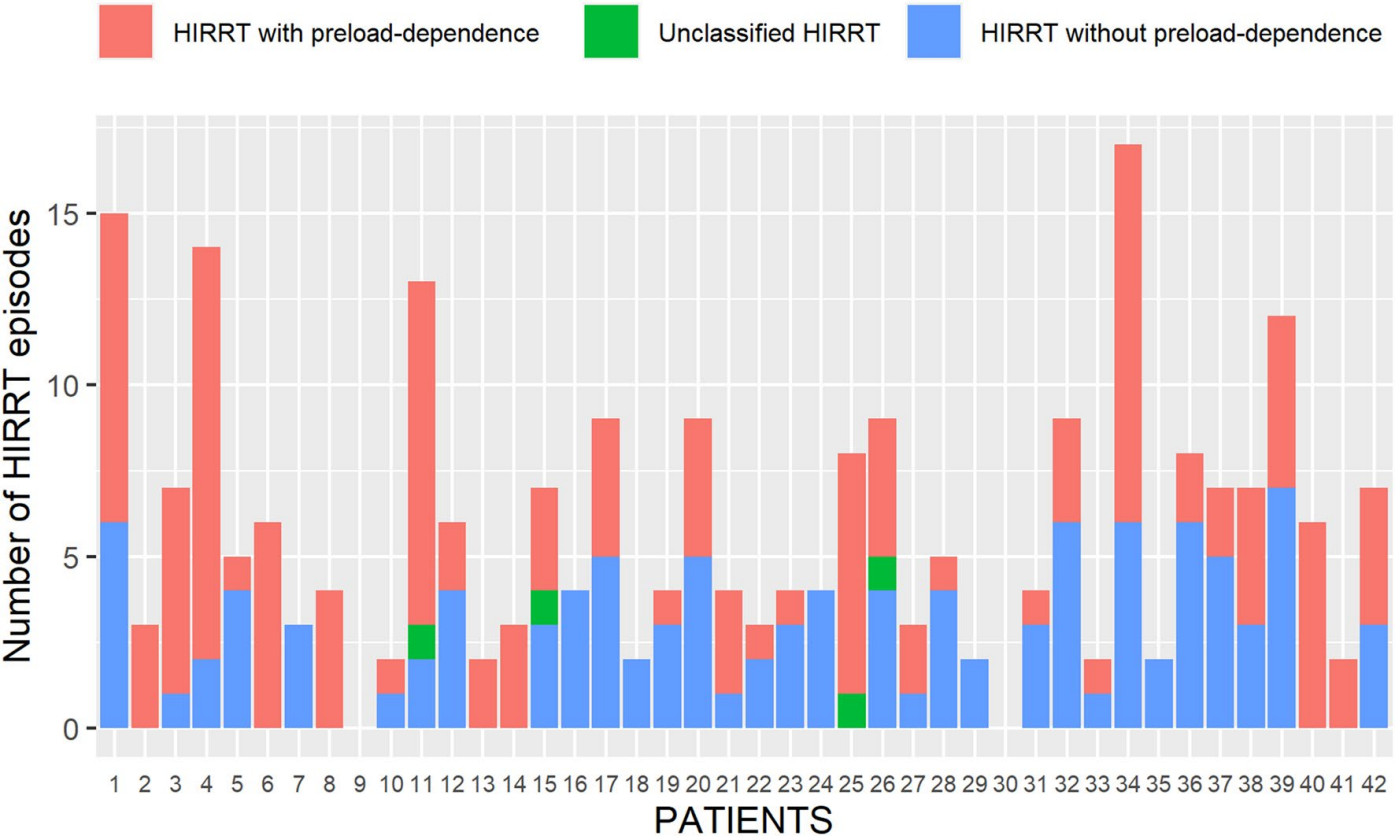
HIRRT episodes as a function of preload-dependence status per patient. *HIRRT* hemodynamic instability related to renal replacement therapy

**Fig. 3:**
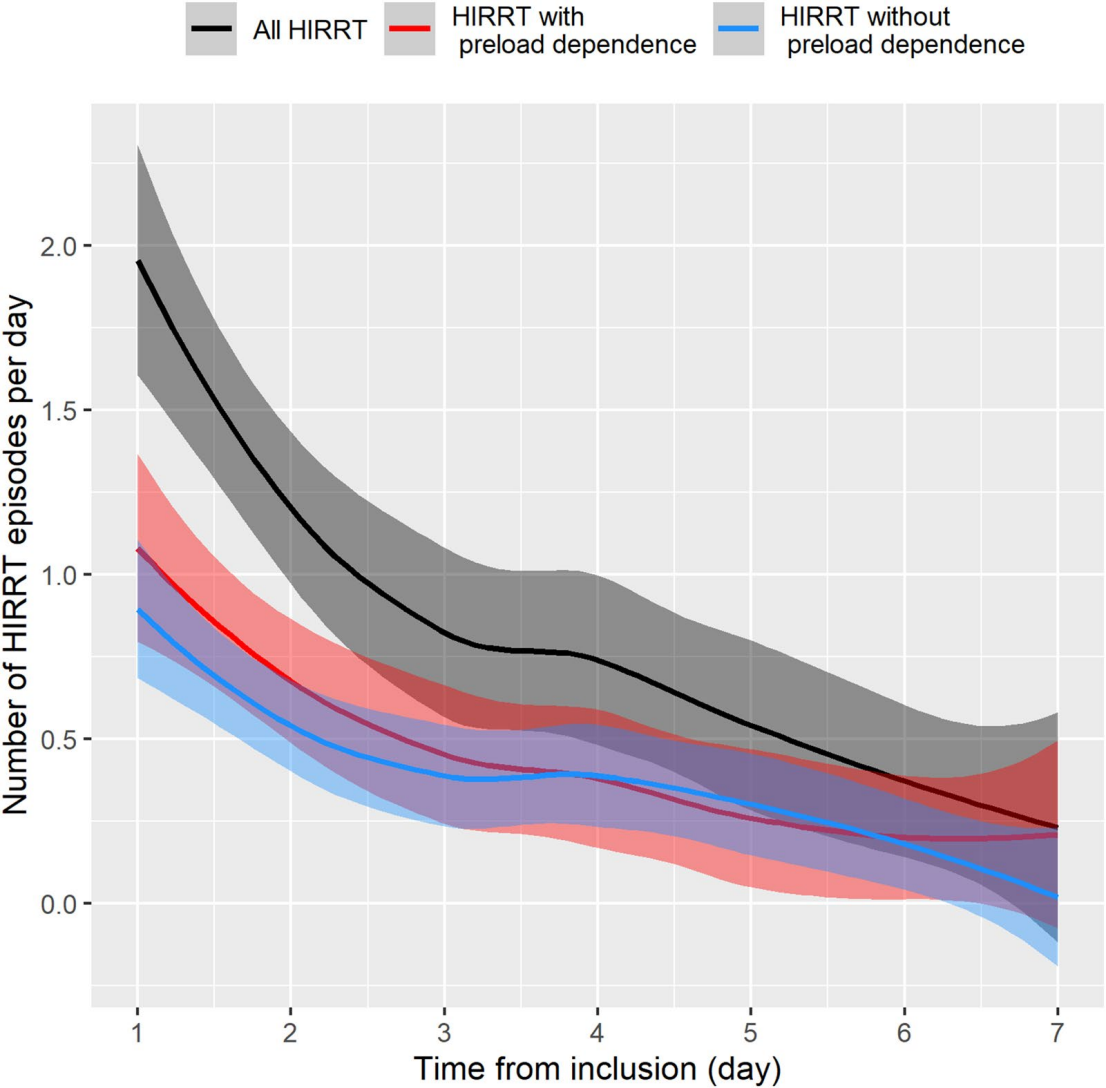
Number of HIRRT episodes per day and per patient following inclusion. Continuous lines are local polynomial regression (LOESS) fits of individual data points. Shaded areas are 95% confidence level interval for predictions. *HIRRT* hemodynamic instability related to renal replacement therapy

**Fig. 4:**
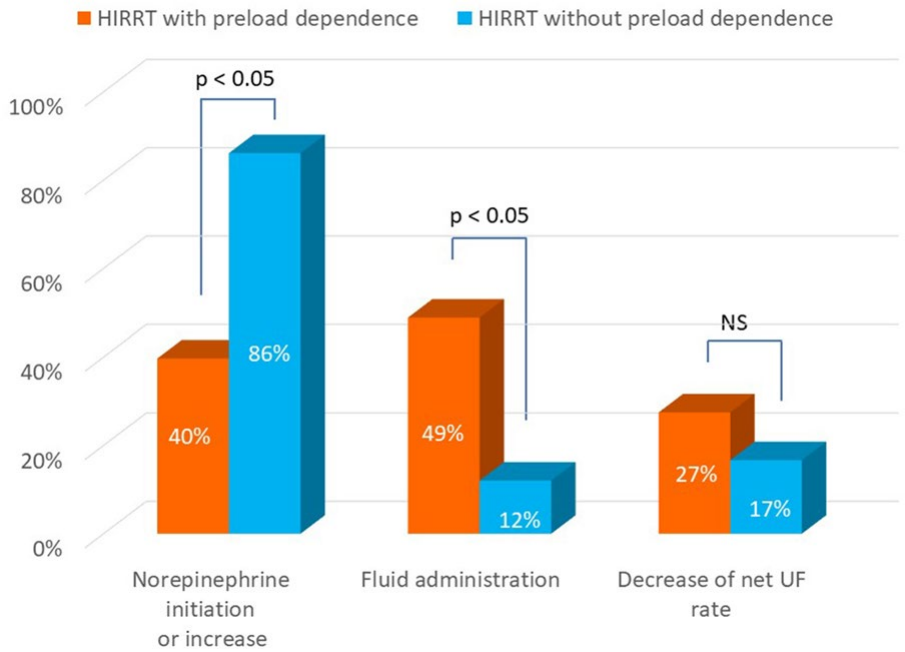
Therapeutic management of HIRRT. Values per type of HIRRT do not add up to 100% since multiple interventions could be selected by attending physician. *HIRRT* hemodynamic instability related to renal replacement therapy, *NS* not significant, *UF* ultrafiltration

Five [3–8] HIRRT episodes occurred per patient, for a pooled total of 243 episodes. Forty patients (98%) experienced at least 1 episode of HIRRT, with most patients experiencing both preload-dependent and preload-independent HIRRT episodes (Fig. 2). One hundred thirty-one HIRRT episodes (54% [CI_95%_ 48–60%]) were associated with preload-dependence, 108 (44%, [CI_95%_ 38–51%]) had no preload-dependence and 4 were unclassified since postural tests were not assessed. The number of both preload-dependent and preload-independent HIRRT episodes per day decreased over time after inclusion (Fig. 3). Therapeutic management of HIRRT episodes differed between, preload and non-preload-dependent HIRRT episodes (Fig. 4). Norepinephrine was more frequently used in HIRRT episodes without preload-dependence while the opposite was true for fluid administration (*p* < 0.05). The delay between the last preceding hemodynamic measurement and HIRRT episode associated with preload-dependence was 104 [61–189] min.

### Risk factors for HIRRT associated with preload-dependence (Fig. 5)

**Fig. 5:**
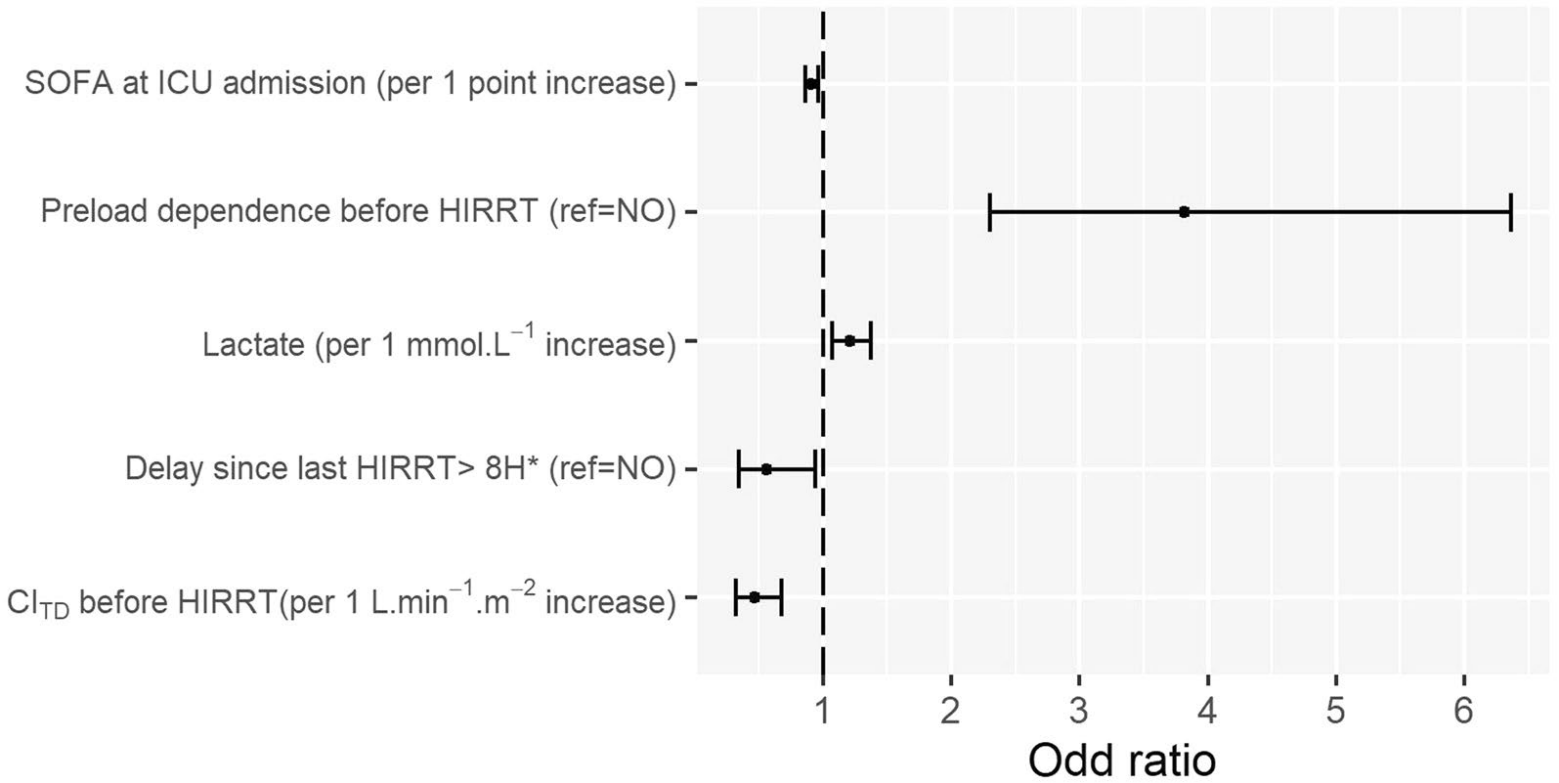
Forest plot of risk factors for occurrence of HIRRT associated with preload-dependence in multivariate analysis. Bars are 95% confidence interval of odds ratios. *CI*_*TD*_ cardiac index assessed by thermodilution, *HIRRT* hemodynamic instability related to renal replacement therapy, *ICU* intensive care unit, *OR* odds ratio. *This cut-off value was chosen as it maximized the Youden’s index in univariate analysis. The following variables were entered into the multivariate full model: preload-dependence on the preceding measurement before HIRRT (yes/no), delay since last HIRRT episode > 8 h (yes/no), delay since CRRT onset, cardiac index assessed by thermodilution on the preceding measurement before HIRRT, global end-diastolic volume on the preceding measurement before HIRRT, pulmonary vascular permeability index on the preceding measurement before HIRRT, pulse pressure variation on the preceding measurement before HIRRT, systolic arterial pressure on the preceding measurement before HIRRT, mechanical ventilation status (yes/no) on the preceding measurement before HIRRT, sex male (yes/no), SOFA score at ICU admission, body weight on the day of hemodynamic measurement, SOFA score on the day of hemodynamic measurement; lactate on the day of hemodynamic measurement, base excess on the day of hemodynamic measurement, hemoglobin on the day of hemodynamic measurement, sepsis criteria on the day of hemodynamic measurement (yes/no), septic shock criteria on the day of hemodynamic measurement (yes/no). The following variables were not entered into the multivariate full model because of multicollinearity: cardiac index assessed by pulse contour analysis and global ejection fraction on the preceding measurement before HIRRT (collinearity with cardiac index assessed by thermodilution), stroke volume variation on the preceding measurement before HIRRT (collinearity with pulse pressure variation), mean and diastolic arterial pressure on the preceding measurement before HIRRT (collinearity with systolic arterial pressure), bicarbonate on the day of hemodynamic measurement (collinearity with base excess). No significant interaction was identified between any of the selected variables. C-statistic of the final model: 0.77. Model calibration assessed by the Hosmer–Lemeshow test: *p* = 0.76

Univariate analysis of variables collected before HIRRT onset as predictors for occurrence of HIRRT associated with preload-dependence is presented in Additional file 3. Multivariate analysis identified the following independent risk factors for occurrence of HIRRT associated with preload-dependence: preload-dependence on the preceding measurement before HIRRT (odds ratio (OR) = 3.82 [2.30–6.36], *p* < 0.001), arterial lactate level on the day of HIRRT (OR = 1.21 [1.07–1.37] per 1 mmol L^−1^ increase, *p* < 0.05), delay since last HIRRT episode of at least 8 h (OR = 0.56 [0.34–0.94], *p* < 0.05), cardiac index assessed by thermodilution on the preceding measurement before HIRRT (OR = 0.47 [0.32–0.68] per 1 L min^−1^ m^−2^ increase, *p* < 0.001) and SOFA score at ICU admission (OR = 0.91 [0.86–0.96] per 1-point increase, *p* < 0.001).

## Discussion

The main findings of the study are the following: (1) HIRRT associated with preload-dependence in patients under CRRT is slightly more frequent than HIRRT without preload-dependence; (2) HIRRT during CRRT is not independently related to CRRT settings in a selected population under invasive continuous hemodynamic monitoring, and is mainly related to underlying cardiovascular dysfunction; (3) preload-dependence is a risk factor for HIRRT associated with preload-dependence during CRRT and monitoring preload-dependence may be useful to adjust net ultrafiltration rate and prevent hemodynamic impairment in ICU patients under CRRT.

To our knowledge, there is no other published study reporting the prevalence of HIRRT associated with preload-dependence during CRRT. We report, with a high granularity of data and over a prolonged period of time, a rate of HIRRT associated with preload-dependence close to 50%, i.e., similar to the rate of preload-dependence during acute circulatory failure [12, 28]. This result suggests that the cessation or reduction of net ultrafiltration rate may not always be an appropriate therapeutic response when facing an HIRRT episode under CRRT. In keeping with this finding, we previously reported that preload-dependence was only present in 19% of HIRRT episodes during intermittent hemodialysis in ICU [11]. Schortgen et al. also reported that HIRRT frequently occurred early during intermittent hemodialysis sessions, prior to the removal of a significant fluid volume via ultrafiltration [29]. These elements emphasize the importance of considering other factors than hypovolemia when facing the life-threatening issue of HIRRT.

Prevalence of HIRRT ranged from 19 to 43% of CRRT treatments in previous observational studies [7, 9, 30]. The variability in the reported frequencies is partly attributable to the lack of a consensus definition of HIRRT. Indeed, Uchino et al. defined HIRRT as a decrease of more than 20 mmHg of systolic blood pressure or any increase of vasopressors whereas Akhoundi et al. defined HIRRT as a new/sudden decrease of systolic blood pressure > 40 mmHg, a mean arterial pressure of < 60 mmHg, or a systolic blood pressure < 90 mmHg, or any initiation or increased dose of vasoactive drugs, or the need for intravenous fluid boluses. In these studies, neither etiology nor mechanisms of HIRRT were investigated and the prevalence of HIRRT associated with preload-dependence was not studied.

However, although the main mechanisms of HIRRT are decreased cardiac output and decreased peripheral resistance, it is well known that HIRRT may be a consequence of multiple other mechanisms in any given patient [31]. These mechanisms include CRRT-related factors (such as modality, ultrafiltration rate and osmolality shift) and patient-related factors (such as myocardial stunning and autonomic dysfunction) [32]. Taken together, these data challenge the notion that HIRRT is predominantly due to excessive ultrafiltration. Thus, it seems that the assessment of preload-dependence or independence status cannot be easily predicted during a HIRRT episode, but requires functional hemodynamic monitoring and continuous cardiac index measurements (as in any hemodynamic instability episode without CRRT). Of note, the use of functional hemodynamic in our study was associated with specific therapeutic interventions as a function of preload-dependence status during HIRRT (Fig. 4), suggesting that it may help personalizing CRRT settings as a function of hemodynamic status. Interestingly, HIRRT with preload-dependence were treated with heterogeneous interventions (i.e., fluid administration, vasopressor, discontinuation or decrease in net ultrafiltration rate), and a comparative study is probably required to evaluate the usefulness of personalized treatment in HIRRT with preload-dependence.

Interestingly, repetitive hemodynamic evaluations during the study (1237 in total) allowed us to identify variables collected before HIRRT onset as independent risk factors for occurrence of HIRRT associated with preload-dependence. Identification of preload-dependence during systematic hemodynamic evaluation before HIRRT onset was a strong predictor of HIRRT associated with preload-dependence. This result is of high importance since contradictory results have been reported about other interventions (sodium profiling, cooler dialysate and UF profiling notably) suggested to prevent HIRRT [31]. Therefore, iterative testing for preload-dependence during CRRT may constitute a useful strategy for guiding net ultrafiltration rate. A higher arterial lactate, a delay since last HIRRT episode below 8 h, and a lower cardiac output were also associated with higher risk of preload-dependence-related HIRRT. These findings were not unexpected since lower cardiac index is expected in preload-dependent patients as a consequence of being in the steep part of the Starling curve [33], arterial lactate is a marker of acute circulatory failure, and lower delay since last HIRRT episode suggests hemodynamic instability. Higher admission SOFA being a protective factor of preload-dependence associated HIRRT may be surprising, although it may be a consequence of more aggressive fluid resuscitation in these patients. We are, however, unable to confirm this hypothesis as the amount of fluid administration was not recorded in the present work. Even if they are not sufficient to predict preload-dependency if assessed alone, those parameters could be analyzed as part of a set of variables to adjust net ultrafiltration rate in patients undergoing CRRT. Surprisingly, the net ultrafiltration rate (nor any other CRRT settings) was not identified as an independent risk factor for occurrence of HIRRT associated with preload-dependence in our study.

This study presents several limits. First, the observational feature precludes drawing any causal associations between the independent variables identified by multivariate analysis and HIRRT associated with preload-dependence. Second, like any single-center study, extrapolation of our results to other ICUs may be questionable. Furthermore, the study population may be highly selected, and it is likely that we studied a population at higher risk for HIRRT since patients without PiCCO monitoring (i.e., considered by attending physicians as less likely to present severe hemodynamic alterations) were excluded by design. Third, the chosen definition of HIRRT could be debated. Indeed, unlike in the context of end-stage kidney disease on maintenance hemodialysis [34], there is no standardized definition of HIRRT during CRRT in ICU patients [31, 32]. We used a pragmatic definition, requiring predefined therapeutic interventions in addition to low arterial pressure to qualify HIRRT, similarly to previous studies [35, 36]. Four, the study stopped prematurely because enrollment was hindered by nurse staff overwork during the COVID-19 pandemics. Nevertheless, the number of HIRRT largely exceeded the required number computed by power analysis. Five, a substantial number of patients were prematurely withdrawn from the study (mainly for death and CRRT cessation), and ICU policy regarding CRRT cessation may have influenced the observed rate and type of HIRRT. Finally, hemodynamic data collection and postural tests were realized by ICU nurses, whose expertise in hemodynamic monitoring may be debatable, although regular training session were organized to ensure quality of data acquisition.

Nevertheless, the study has the following strengths. First, the prospective feature of the study ensured a very low rate of missing values, which were nevertheless taken into account during statistical analysis. Second, the high number of HIRRT episodes allowed a high number of risk factors for HIRRT to be selected for inclusion in the multivariate model. Third, preload-dependence was assessed with both PLR in the supine position and the Trendelenburg maneuver in the prone position, i.e., two techniques with very high diagnostic performance to identify preload-dependency [12, 13]. Furthermore, it has been demonstrated that renal replacement therapy does not alter the measurement of cardiac index by transpulmonary thermodilution and pulse contour analysis [37, 38]. Fourth, although requiring multiple complex hemodynamic evaluations over the first 7 days after inclusion, the study demonstrate the feasibility of this monitoring strategy in real-life, without additional nursing staff.

## Conclusions

In this single-center study, HIRRT associated with preload-dependence was slightly more frequent than HIRRT without preload-dependence in ICU patients undergoing CRRT. Iterative testing for preload-dependence could help avoiding unnecessary decrease in fluid removal during CRRT, but this must be confirmed in interventional controlled trials.

## Supplementary Information

**Additional file 1: Table S1**. Description of data: missing values per variable.

**Additional file 2: Figure S1.** Description of data: percentage of patients remaining in study during the first 7 days after inclusion.

**Additional file 3: Table S2.** Description of data: risk factors for occurrence of HIRRT associated with preload-dependence in univariate analysis.

## Data Availability

The datasets used and/or analyzed during the current study are available from the corresponding author on reasonable request.

## Abbreviations

AKI: Acute kidney injury
CI_95%_: Ninety-five percent confidence interval
CRRT: Continuous renal replacement therapy
CVVH: Continuous veno-venous hemofiltration
CVVHD: Continuous veno-venous hemodialysis
HIRRT: Hemodynamic instability related to renal replacement therapy
ICU: Intensive care unit
OR: Odds ratio
PLR: Passive leg raising
PPV: Pulse pressure variation
SAPS: Simplified Acute Physiology Score
SOFA: Sequential Organ Failure Assessment
SVV: Stroke volume variation
