# Supplementary material for: Prevalence and risk factors of hemodynamic instability associated with preload-dependence during continuous renal replacement therapy in a prospective observational cohort of critically ill patients
Source: Ann Intensive Care. 2021 Jun 14;11:95. doi: 10.1186/s13613-021-00883-9 (PMC8200783; doi:10.1186/s13613-021-00883-9)
Supplement: Supplementary file 1 — Additional file 1: Table S1. Description of data: missing values per variable. [file 13613_2021_883_MOESM1_ESM.docx]

Additional file 1: Table S1. Missing values per variable.

| Variables | Count (%) |
| --- | --- |

| ***Data at ICU admission*** |
| --- |

| Age | 0 (0%) |
| --- | --- |
| Sex | 0 (0%) |
| SAPS 2 | 0 (0%) |
| SOFA | 0 (0%) |
| Admission category | 0 (0%) |

| ***Data at inclusion*** |
| --- |

| Height at inclusion | 0 (0%) |
| --- | --- |
| Weight at inclusion | 0 (0%) |
| SOFA at inclusion | 0 (0%) |
| Sepsis * at inclusion | 0 (0%) |
| Septic shock * at inclusion | 0 (0%) |

| ***Data assessed daily*** |  |
| --- | --- |

| Daily weight | 16 (9%) |
| --- | --- |
| Daily coagulation-SOFA subscore | 6 (3%) |
| Daily hepatic-SOFA subscore | 27 (16%) |
| Daily renal-SOFA subscore | 3 (2%) |
| Daily respiratory-SOFA subscore | 3 (2%) |
| Daily cardiovascular-SOFA subscore | 1 (1%) |
| Daily neurologic-SOFA subscore | 1 (1%) |
| Daily global SOFA score | 27 (16%) |
| Daily PaO_2_/FiO_2_ | 4 (2%) |
| Daily pH | 2 (1%) |
| Daily PaCO_2_ | 3 (2%) |
| Daily bicarbonates | 3 (2%) |
| Daily base excess | 3 (2%) |
| Daily lactate | 22 (13%) |
| Daily hemoglobin | 4 (2%) |
| Daily Sepsis * | 0 (0%) |
| Daily Septic shock * | 0 (0%) |

| ***Data assessed at each hemodynamic evaluation*** |  |
| --- | --- |

| Time between CRRT onset and hemodynamic evaluations (hr) | 0 (0%) |
| --- | --- |
| Time between inclusion and hemodynamic evaluations (hr) | 0 (0%) |
| Type of CRRT | 0 (0%) |
| CRRT circuit anticoagulation | 0 (0%) |
| Ultrafiltrate/dialysate temperature (°C) | 15 (1%) |
| Net UF (ml.hr^-1^) | 1 (0%) |
| Blood flow (ml.min^-1^) | 1 (0%) |
| Ultrafiltration rate (ml.kg^-1^.hr^-1^) ** | 0 (0%) |
| Dialysate rate (ml.hr^-1^) *** | 0 (0%) |
| CI_PC_ (L.min^-1^.m^-2^) | 31 (3%) |
| Preload dependence assessed by postural test | 37 (3%) |
| PPV (%) | 75 (6%) |
| SVV (%) | 185 (15%) |
| Ea_dyn_ | 188 (15%) |
| CI_TD_ (L.min^-1^.m^-2^) | 62 (5%) |
| ISVR (dynes.sec.cm^-5^) | 95 (8%) |
| GEF (%) | 75 (6%) |
| EVLWI (ml.kg^-1^) | 66 (5%) |
| GEDVI (mL.m^-2^) | 71 (6%) |
| PVPI | 70 (6%) |
| Vasopressor dose (µg.kg^-1^.min^-1^) | 5 (0%) |
| Dobutamine dose (µg.kg^-1^.min^-1^) | 0 (0%) |
| SAP (mm Hg) | 10 (1%) |
| MAP (mm Hg) | 10 (1%) |
| DAP (mm Hg) | 10 (1%) |
| Heart rate (min^-1^) | 8 (1%) |
| CVP (mm Hg) | 34 (3%) |
| Position | 0 (0%) |
| Mechanical ventilation | 0 (0%) |
| Preload dependence assessed during HIRRT episode | 4 (0%) |

BMI = body mass index; SAPS 2 = simplified Acute Physiology Score 2; SOFA = sequential Organ Failure Assessment; CRF = chronic respiratory failure; CHF = chronic heart failure; ICU = intensive care unit; CRRT = continuous renal replacement therapy; HIRRT = hemodynamic instability related to renal replacement therapy; UF = ultrafiltration; CI_PC_ = cardiac index assessed by pulse contour analysis; CVP = central venous pressure; delta CVP = CVP (mm Hg) at postural test onset - CVP (mm Hg) at postural test outset; PPV = pulse pressure variation; SVV = stroke volume variation; Ea_dyn_ = dynamic arterial elastance; CI_TD_ = cardiac index assessed by thermodilution; ICU = intensive care unit; ISVR = indexed systemic vascular resistance; GEF = global ejection fraction; EVLWI = extravascular lung water index; GEDVI = global end-diastolic volume index; PVPI = pulmonary vascular permeability index; SAP = systolic arterial pressure; MAP = mean arterial pressure; DAP = diastolic arterial pressure; PEEP = positive end-expiratory pressure.

* According to sepsis 3 criteria (27), ** in patients treated with CVVH (continuous veno-venous hemofiltration), *** in patients treated with CVVHD (continuous veno-venous hemodialysis).
