# Supplementary material for: Prevalence and risk factors of hemodynamic instability associated with preload-dependence during continuous renal replacement therapy in a prospective observational cohort of critically ill patients
Source: Ann Intensive Care. 2021 Jun 14;11:95. doi: 10.1186/s13613-021-00883-9 (PMC8200783; doi:10.1186/s13613-021-00883-9)

Additional file 2: Figure S1. Percentage of patients remaining in study during the first seven days after inclusion


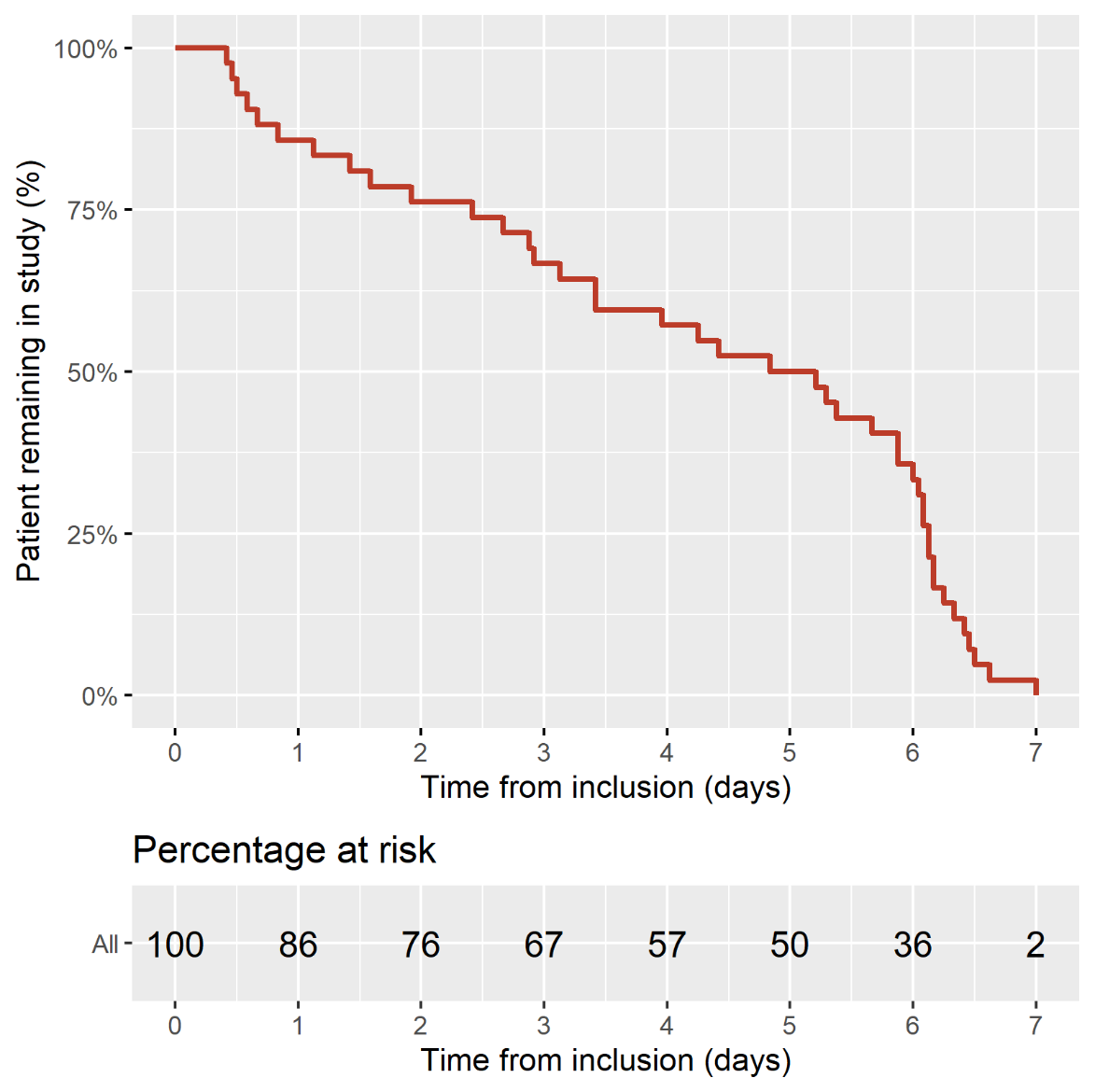

Supplement: Supplementary file 2 — Additional file 2: Figure S1. Description of data: percentage of patients remaining in study during the first 7 days after inclusion. [file 13613_2021_883_MOESM2_ESM.docx]
