# Supplementary material for: Prevalence and risk factors of hemodynamic instability associated with preload-dependence during continuous renal replacement therapy in a prospective observational cohort of critically ill patients
Source: Ann Intensive Care. 2021 Jun 14;11:95. doi: 10.1186/s13613-021-00883-9 (PMC8200783; doi:10.1186/s13613-021-00883-9)
Supplement: Supplementary file 3 — Additional file 3: Table S2. Description of data: risk factors for occurrence of HIRRT associated with preload-dependence in univariate analysis. [file 13613_2021_883_MOESM3_ESM.docx]

Additional file 3: Table S2. Risk factors for the occurrence of HIRRT associated with preload-dependence in univariate analysis.

| Variables | Odd ratio [CI_95%_] | p value |
| --- | --- | --- |
| *Collected at ICU admission* |  |  |
| Age (yr) | 1.01 [0.98-1.04] | p=0.58 |
| Sex male | 1.89 [0.87-4.49] | **p=0.10** |
| Body weight at ICU admission (kg) | 0.99 [0.97-1.01] | p=0.27 |
| SAPS2 at ICU admission | 0.99 [0.97-1.02] | p=0.53 |
| SOFA at ICU admission | 0.92 [0.85-0.99] | **p<0.05** |
| *Collected at inclusion* |  |  |
| Sepsis at inclusion* (ref=No) | 1.89 [0.73-5.90] | **p=0.19** |
| Septic shock at inclusion* (ref=No) | 1.49 [0.69-3.69] | p=0.32 |
| *Collected on measurement day* |  |  |
| Body weight on measurement day (kg) | 0.98 [0.96-1.01] | **p=0.18** |
| Fluid balance on measurement day (kg) | 1.00 [0.95-1.04] | p=0.97 |
| SOFA score on measurement day | 1.08 [0.97-1.20] | **p=0.14** |
| PaO_2_/FiO_2_ on measurement day (mm Hg) | 1.00 [0.99-1.00] | p=0.22 |
| pH on measurement day | 0.24 [0.01-4.81] | p=0.34 |
| PaCO_2_ on measurement day (mm Hg) | 0.99 [0.96-1.02] | p=0.48 |
| Bicarbonates on measurement day (mmol.L^-1^) | 0.93 [0.86-0.99] | **p<0.05** |
| Base excess on measurement day (mmol.L^-1^) | 0.94 [0.88-1.00] | **p=0.06** |
| Lactate on measurement day (mmol.L^-1^) | 1.21 [1.06-1.38] | **p<0.01** |
| Hemoglobin on measurement day (g.L^-1^) | 1.02 [1.00-1.04] | **p<0.05** |
| Sepsis criteria on measurement day* (ref=No) | 2.46 [0.93-8.32] | **p=0.07** |
| Septic shock criteria on measurement day* (ref=No) | 2.09 [1.19-3.74] | **p<0.05** |
| *Collected on the measurement immediately preceding HIRRT* | | |
| Preload dependency (ref=No) | 2.95 [1.80-4.87] | **p<0.001** |
| Delay since CRRT onset (hr) | 0.99 [0.99-1.00]** | **p<0.05** |
| Delay since last HIRRT episode >8 hr (ref=No) | 0.60 [0.37-0.98] | **p<0.05** |
| Net ultrafiltration rate (ml.kg.hr^-1^) | 0.87 [0.68-1.10] | p=0.24 |
| Ultrafiltrate/dialysate temperature(°C) | 1.01 [0.77-1.31] | p=0.96 |
| CRRT blood flow (mL.min^-1^) | 1.00 [0.99-1.00] | p=0.37 |
| Ultrafiltrate or dialysate rate (ml.kg.hr^-1^) | 1.01 [0.96-1.06] | p=0.62 |
| PPV (%) | 1.04 [1.00-1.08] | **p=0.07** |
| SVV (%) | 1.03 [0.99-1.06] | **p=0.17** |
| Ea_dyn_ | 0.77 [0.35-1.62] | p=0.49 |
| CI_PC_(L.min^-1^.m^-2^) | 0.40 [0.28-0.58] | **p< 0.001** |
| CI_TD_ (L.min^-1^.m^-2^) | 0.45 [0.30-0.66] | **p<0.001** |
| GEF (%) | 0.94 [0.89-0.99] | **p<0.05** |
| EVLWI (ml.kg^-1^ PBW) | 0.99 [0.91-1.08] | p=0.83 |
| GEDVI (mL.m^-2^) | 1.00 [1.00-1.00] | **p=0.16** |
| PVPI | 1.36 [0.90-2.06] | **p=0.15** |
| Norepinephrine dose (µg.kg^-1^.min^-1^) | 1.01 [0.76-1.29] | p=0.97 |
| Norepinephrine administration (ref=No) | 1.23 [0.58-2.71] | p=0.59 |
| Dobutamine administration (ref=No) | 1.39 [0.44-4.04] | p=0.55 |
| Systolic arterial pressure (mm Hg) | 0.99 [0.98-1.00] | **p=0.11** |
| Mean arterial pressure (mm Hg) | 0.97 [0.95-1.00]** | **p<0.05** |
| Diastolic arterial pressure (mm Hg) | 0.99 [0.96-1.01] | p=0.29 |
| Heart rate (min^-1^) | 1.00 [0.99-1.01] | p=0.90 |
| CVP (mm Hg) | 1.02 [0.95-1.09] | p=0.60 |
| Mechanical ventilation (ref=No) | 3.65 [1.12-16.8] | **p<0.05** |
| PEEP (cm H_2_O) | 1.00 [0.91-1.10] | p=0.92 |
| Respiratory rate (min^-1^) | 1.02 [0.98-1.07] | p=0.41 |
| VT (mL.kg^-1^ PBW) | 0.90 [0.72-1.11] | p=0.33 |

CI_95%_ = 95% confidence interval; CI_PC_ = cardiac index assessed by pulse contour analysis; CI_TD_ = cardiac index assessed by thermodilution; CRRT = continuous renal replacement therapy; CVP = central venous pressure; CVVH = continuous veno-venous hemofiltration; CVVHD = continuous veno-venous hemodialysis; Ea_dyn_ = dynamic arterial elastance; EVLWI = extravascular lung water index; FiO_2_ = fraction of inspired oxygen; GEDVI = global end-diastolic volume index; GEF = global ejection fraction; HIRRT = hemodynamic intolerance related to renal replacement therapy; ICU = intensive care unit; MAP = mean arterial pressure; PaCO_2_ = carbon dioxide partial pressure in arterial blood; PaO_2_ = oxygen partial pressure in arterial blood; PBW = predicted body weight; PEEP = positive end-expiratory pressure; PPV = pulse pressure variation; PVPI = pulmonary vascular permeability index; SAPS 2 = simplified Acute Physiology Score 2; SOFA = sequential Organ Failure Assessment; SVV = stroke volume variation; VT=tidal volume.

* According to sepsis 3 criteria. ** upper value of the CI_95%_  is lower than 1 at a precision lower than 2 digits after the decimal point.
